# Clinicopathological and epidemiological significance of breast cancer subtype reclassification based on p53 immunohistochemical expression

**DOI:** 10.1038/s41523-019-0117-7

**Published:** 2019-07-25

**Authors:** Mustapha Abubakar, Changyuan Guo, Hela Koka, Hyuna Sung, Nan Shao, Jennifer Guida, Joseph Deng, Mengjie Li, Nan Hu, Bin Zhou, Ning Lu, Xiaohong R. Yang

**Affiliations:** 1 Division of Cancer Epidemiology and Genetics, National Cancer Institute, NIH, DHHS, Bethesda, MD 20892 USA; 2 National Cancer Center/Cancer Hospital, Chinese Academy of Medical Sciences and Peking Union Medical College, 100021 Beijing, China; 3 Surveillance and Health Services Research, American Cancer Society, Atlanta, GA 30303 USA; 4 Department of Breast and Thyroid Surgery, The First Affiliated Hospital of Sun Yat-sen University, 510275 Guangzhou, China; 5 Division of Cancer Control and Population Sciences, National Cancer Institute, NIH, DHHS, Bethesda, MD 20892 USA; 6 Vanderbilt University, Nashville, TN 37235 USA

**Keywords:** Breast cancer, Cancer epidemiology, Prognostic markers

## Abstract

*TP53* mutations are common in breast cancer and are typically associated with more aggressive tumor characteristics, but little is known about the clinicopathological and epidemiological relevance of p53 protein expression, a *TP53* mutation surrogate, in breast cancer subtypes. In this study of 7226 Chinese women with invasive breast cancer, we defined breast cancer subtypes using immunohistochemical (IHC) measures of hormone receptors and HER2 in conjunction with histologic grade. p53 expression status was then used to further stratify subtypes into p53-positive and p53-negative. Odds ratios (ORs) and 95% confidence intervals (CIs) in case-only logistic regression analyses were used to examine heterogeneity across different subtypes. The frequency of p53 protein expression varied by breast cancer subtype, being lowest in the luminal A-like and highest in the triple-negative and HER2-enriched subtypes (*P*-value < 0.01). In luminal A-like and B-like/HER2-negative subtypes, p53 positivity was associated with early-onset tumors, high grade, high proliferative index, and basal marker (CK5/6 and EGFR) expression. Further, compared with luminal A-like/p53-negative patients, A-like/p53-positive patients were more likely to be parous [adjusted OR _parous vs. nulliparous_ = 2.67 (1.60, 4.51); *P*-value < 0.01] and to have breastfed [adjusted OR _ever vs. never_ = 1.38 (1.03, 1.85); *P*-value = 0.03]. p53 positivity was not associated with examined clinical and risk factors in other tumor subtypes. Overall, these findings suggest that p53 expression, which is readily available in many settings, can be used to identify phenotypes of luminal A-like breast cancer with distinct clinical and epidemiological implications.

## Introduction

Based on gene-expression profiling, breast cancer can be classified into luminal, human epidermal growth factor receptor 2 (HER2)-enriched, normal-like, and basal-like subtypes,^1^ with distinct clinical and epidemiological attributes.^2–4^ In general, compared to non-luminal (HER2-enriched and basal-like) breast cancers, luminal tumors have better survival outcomes and are more strongly associated with reproductive risk factors.^5–7^ Nonetheless, accumulating evidence is in support of residual clinical and epidemiological heterogeneity within the major breast cancer subtypes.^8^ This is particularly relevant for luminal breast cancer, which constitutes the majority of breast cancers and is characterized by substantial diversity in molecular characteristics and clinical outcomes.^4,9–11^

Despite its relatively good prognosis overall,^2,12^ some patients with the luminal breast cancer subtype suffer from fatal recurrence on endocrine therapy.^9,13^ Expression profiling studies have identified at least two subtypes of luminal breast cancer, designated as luminal A and B, with different prognoses and response to treatment.^4,14,15^ To recapitulate these subtypes in routine clinical practice, some international guidelines have endorsed the use of immunohistochemical measures on estrogen receptor (ER), progesterone receptor (PR), HER2 and KI67, a marker of proliferation, or histologic grade, a composite marker of proliferation and differentiation.^16–18^ Although this classification scheme has been shown to be of prognostic^19^ and therapeutic^16,20^ relevance, it does not fully capture heterogeneity in luminal tumors.^21,22^

The *TP53* gene is the most commonly mutated gene in human cancers and functions in many cellular pathways including cell cycle regulation, metabolism, angiogenesis, and DNA repair mechanisms.^23,24^ Approximately 30% of breast tumors are believed to harbor mutations in *TP53*,^25^ and recent data suggested that the frequency, spectrum, and timing of these mutations varied according to the molecular subtype of the disease.^26–29^ In general, *TP53* mutations were less common in luminal (A and B) than basal-like tumors; occurring in 26% of luminal tumors (17% for luminal A and 41% for luminal B) as compared to 50% of HER2-enriched and 88% of basal-like breast tumors.^27,28^ Further, unlike basal-like tumors in which *TP53* mutation is believed to be a late event that occurs only after *PTEN* loss, *TP53* mutation is thought to be an early event in luminal tumors.^26,28^ The physicochemical properties of the protein produced by the mutant *TP53*, including its non-biodegradability and its stable accumulation in the cell nuclei,^30^ are different from the wildtype p53, which forms the basis for its assessment using IHC. Strong and diffuse immunostaining of p53 is generally interpreted as likely to have a *TP53* gene mutation.

It has previously been shown that differences in *TP53* mutation, as well as p53 protein expression, between breast cancer subtypes may confer variability in clinical behavior within these subtypes.^29,31,32^ However, to the best of our knowledge, the epidemiological relevance of p53 protein expression in molecular subtypes of breast cancer is yet to be evaluated in any previous study. Our aim in this study was, therefore, to perform a large-scale evaluation of the clinical and epidemiological significance of p53 protein expression, as a surrogate for *TP53* mutation status, in molecular subtypes of breast cancer.

## Results

### Patient characteristics by p53 expression status

Overall, 7226 women were included in this analysis. The mean age at diagnosis was 51.6 years (standard deviation = 10.8). Compared with women with p53− tumors, those with p53+ tumors were generally younger (*P*-value = 0.01) and had higher frequencies of aggressive tumor features including poorly differentiated (*P*-value = 1.06 × 10^−26^), large size (*P*-value = 0.002), more proliferative (higher frequency of KI67+; *P*-value = 1.96 × 10^−35^), CK5/6+ (*P*-value = 4.31 × 10^−11^), EGFR+ (*P*-value = 3.55 × 10^−20^), and aggressive molecular subtypes (B-like/HER2−, B-like/HER2+, HER2-enriched and TN; *P*-value = 7.21 × 10^−30^) (Table 1). In general, the prevalence of p53 IHC positive staining differed by subtype, with luminal A-like tumors comprising of fewer (46%) p53+ tumors than women with the luminal B-like/HER2− (58%), luminal B/HER2+ (60%), TN (61%), and HER2-enriched (63%) subtypes (*P*-value < 0.001). We did not observe differences in the distributions of breast cancer risk factors, including age at menarche, parity, breastfeeding, body mass index (BMI) and family history, by p53 expression status (Table 2).

**Table 1:** Overall distribution of tumor clinicopathological features by p53 expression

Characteristic	p53-		p53+		P*
	No.	%	No.	%	
Age (years)					
<40	392	11.5	453	11.8	
40–50	1132	33.3	1327	34.6	
50–60	1067	31.4	1259	32.8	
60–70	551	16.2	581	15.2	
70+	251	7.4	213	5.6	0.01
Histologic grade					
Well differentiated	262	8.4	144	4.1	
Moderately differentiated	1984	64.1	2014	57.7	
Poorly differentiated	846	27.3	1333	38.2	1.06E−26
Missing	301		342		
Tumor size					
<2 cm	702	48.9	703	42.7	
2–5 cm	694	48.3	885	53.8	
>5 cm	40	2.8	58	3.5	0.002
Missing	1957		2187		
Number of positive lymph nodes					
0	1687	52.4	1914	52.1	
1–3	900	27.9	980	26.7	
>3	634	19.7	777	21.2	0.24
Missing	172		162		
ER status					
Negative	646	19.1	1088	28.4	
Positive	2745	80.9	2743	71.6	1.64E−20
Missing	2		2		
PR status					
Negative	786	23.2	1141	29.8	
Positive	2604	76.8	2688	70.2	2.31E−10
Missing	3		4		
HER2 status					
Negative	2795	82.5	2882	75.3	
Positive	593	17.5	946	24.7	8.53E−14
Missing	5		5		
KI67 (%)					
Q1 (<10)	669	19.8	434	11.4	
Q2 (10–20)	866	25.7	779	20.4	
Q3 (20–35)	1103	32.7	1367	35.8	
Q4 (>35)	737	21.8	1233	32.4	3.68E−39
Missing	18		20		
CK5/6 status					
Negative	2387	90.9	2410	85.1	
Positive	239	9.1	423	14.9	4.31E−11
Missing	767		1000		
EGFR status					
Negative	2050	78.8	1900	67.7	
Positive	550	21.2	905	32.3	3.55E−20
Missing	793		1028		
Molecular subtype					
A-like	1737	54.3	1475	40.2	
B-like/HER2−	528	16.6	726	19.8	
B-like/HER2+	394	12.3	600	16.4	
HER2-enriched	199	6.2	346	9.4	
TNBC	337	10.6	522	14.2	7.21E−30

*P value for chi-square test

### Clinicopathologic significance of p53 expression in subtypes

Table 3 shows the associations between p53 expression and tumor clinicopathological factors within each major breast cancer subtype. We observed significant heterogeneity in age associations across different subtypes (*P*-value for heterogeneity (P-het) = 0.001). While p53 positivity was associated with decreasing age among luminal A-like patients (adjusted OR_trend_ = 0.89 (0.81, 0.99); P-trend = 0.002), it was associated with increasing age among HER2-enriched patients (adjusted OR_trend_ = 1.22 (1.01, 1.47); P-trend = 0.03). In general, associations between p53 status and clinicopathological factors were similar for luminal A-like and luminal B-like/HER2− subtypes, in which p53 positivity was associated with more aggressive tumor features such as higher grade, higher levels of proliferation (indicated by KI67 positivity), and higher frequencies of positive staining for basal markers (CK5/6 and EGFR). There was no consistent pattern of associations between p53 expression and tumor clinicopathological characteristics among other breast cancer subtypes (Table 3).

**Table 2:** Overall distribution of epidemiological risk factors by p53 expression

Characteristic	p53−		p53+		P*
	No.	%	No.	%	
Age at Menarche					
≤12 years	238	10.4	247	10.3	
13 years	397	17.3	460	19.2	
14 years	488	21.2	523	21.8	
≥15 years	1176	51.1	1168	48.7	0.27
Missing	1094		1435		
Parity					
None	108	4.7	89	3.8	
1	1344	58.6	1426	60	
2	612	26.7	609	25.6	
≥3	229	10.0	253	10.6	0.27
Missing	1100		1456		
Breastfeeding					
Never	270	13.3	259	13.2	
Ever	1764	86.7	1704	86.8	0.94
Missing	269		434		
BMI (kg/m^2^)					
<18.5	46	2.0	57	2.4	
18.5–24.99	1213	52.3	1315	54.8	
25–30	853	36.7	825	34.4	
>30	209	9.0	201	8.4	0.20
Missing	1072		1435		
Family history					
Absent	2175	93.0	2262	92.8	
Present	164	7.0	175	7.2	0.82
Missing	1054		1396		

*P value for chi-square test

### Epidemiological significance of p53 expression in subtypes

Table 4 shows the associations between p53 expression and established breast cancer risk factors within each molecular subtype. Among luminal A-like women, we observed that p53+ patients were more likely to be parous [adjusted OR (95% CI) = 2.67 (1.59, 4.51), 2.63 (1.52, 4.55), 3.67 (2.01, 6.71) for 1, 2, and 3 children, respectively, vs. nulliparity; P-trend = 0.006] and have had breastfed [adjusted OR (95% CI) = 1.38 (1.03, 1.85) for ever vs. never; *P*-value = 0.03] (Table 4). Other risk factors, including age at menarche, BMI, and family history of breast cancer, did not differ significantly by p53 status among luminal A-like patients overall. Further, none of the examined risk factors was associated with p53 expression in the other breast cancer subtypes (Table 4).

**Table 3:** Adjusted odds ratios and 95% confidence intervals for the associations between tumor clinicopathological features and subtypes of breast cancer re-classified based on p53 expression

Characteristic	A-like		B-like/HER2−		B-like/HER2+		HER2-enriched		TNBC	
	p53+ vs. p53−		p53+ vs. p53−		p53+ vs. p53−		p53+ vs. p53−		p53+ vs. p53−	
	OR (95% CI)	*P*	OR (95% CI)	*P*	OR (95% CI)	*P*	OR (95% CI)	*P*	OR (95% CI)	*P*
Age (years)										
<40	1.00 (reference)		1.00 (reference)		1.00 (reference)		1.00 (reference)		1.00 (reference)	
40–50	1.13 (0.88, 1.46)	0.32	0.92 (0.62, 1.39)	0.71	1.25 (0.83, 1.88)	0.28	1.67 (0.90, 3.10)	0.10	0.91 (0.56, 1.48)	0.72
50–60	0.88 (0.67, 1.15)	0.36	0.94 (0.63, 1.40)	0.76	1.25 (0.83, 1.89)	0.29	2.16 (1.20, 3.90)	0.01	1.13 (0.70, 1.85)	0.61
60–70	0.85 (0.64, 1.13)	0.27	1.08 (0.69, 1.70)	0.72	1.32 (0.79, 2.20)	0.28	2.00 (0.98, 4.09)	0.06	1.32 (0.74, 2.34)	0.35
70+	0.74 (0.52, 1.06)	0.11	0.99 (0.55, 1.81)	0.98	1.11 (0.54, 2.25)	0.78	2.08 (0.74, 5.82)	0.16	0.65 (0.33, 1.29)	0.22
*P* *trend*		<0.01		0.26		0.98		0.03		
Histologic grade*										
Well diff.	1.00 (reference)		1.00 (reference)		1.00 (reference)				1.00 (reference)	
Mod. diff.	1.40 (1.10, 1.77)	<0.01	4.79 (0.98, 23.52)	0.05	0.33 (0.07, 1.61)	0.17	1.00 (reference)		0.26 (0.02, 3.08)	0.29
Poorly diff.	NE		5.33 (1.09, 25.99)	0.03	0.34 (0.07, 1.66)	0.18	1.18 (0.78, 1.77)	0.42	1.15 (0.80, 1.64)	0.46
Tumor size										
<2 cm	1.00 (reference)		1.00 (reference)		1.00 (reference)		1.00 (reference)		1.00 (reference)	
2–5 cm	0.99 (0.78, 1.24)	0.92	1.10 (0.75, 1.62)	0.61	0.92 (0.60, 1.42)	0.71	1.37 (0.74, 2.54)	0.31	1.10 (0.71, 1.68)	0.67
>5 cm	0.96 (0.47, 1.96)	0.92	2.91 (0.98, 8.65)	0.05	3.12 (0.62, 15.66)	0.17	0.32 (0.07, 1.43)	0.13	0.95 (0.32, 2.77)	0.93
*P* *trend*		0.84		0.08		0.87		0.90		
Lymph nodes										
0	1.00 (reference)		1.00 (reference)		1.00 (reference)		1.00 (reference)		1.00 (reference)	
1–3	0.95 (0.80, 1.12)	0.56	1.00 (0.75, 1.34)	0.99	0.92 (0.67, 1.27)	0.62	0.80 (0.51, 1.26)	0.34	0.74 (0.52, 1.05)	0.09
>3	1.09 (0.89, 1.34)	0.38	0.98 (0.72, 1.33)	0.89	0.95 (0.68, 1.32)	0.75	0.89 (0.56, 1.40)	0.61	0.81 (0.54, 1.05)	0.30
KI67 (%)										
Q1 (<10)	1.00 (reference)		1.00 (reference)		1.00 (reference)		1.00 (reference)		1.00 (reference)	
Q2 (10–20)	1.33 (1.09, 1.63)	<0.01	1.37 (0.83, 2.26)	0.22	1.37 (0.77, 2.43)	0.28	1.68 (0.67, 4.22)	0.27	1.04 (0.43, 2.52)	0.92
Q3 (20–35)	1.81 (1.48, 2.21)	<0.01	2.09 (1.32, 3.32)	<0.01	1.46 (0.87, 2.45)	0.16	2.09 (0.91, 4.80)	0.08	0.51 (0.23, 1.14)	0.10
Q4 (>35)	2.43 (1.84, 3.20)	<0.01	2.74 (1.71, 4.39)	<0.01	1.69 (0.98, 2.91)	0.06	2.22 (0.96, 5.16)	0.06	0.53 (0.24, 1.17)	0.12
*P* *trend*		<0.01		<0.01		0.26		0.02		
CK5/6 status										
Negative	1.00 (reference)		1.00 (reference)		1.00 (reference)		1.00 (reference)		1.00 (reference)	
Positive	1.06 (0.55, 2.03)	0.86	1.74 (1.10, 2.75)	0.02	1.31 (0.64, 2.68)	0.46	0.78 (0.41, 1.48)	0.45	1.18 (0.84, 1.66)	0.34
EGFR status										
Negative	1.00 (reference)		1.00 (reference)		1.00 (reference)		1.00 (reference)		1.00 (reference)	
Positive	1.80 (1.27, 2.54)	<0.01	1.05 (0.73, 1.51)	0.79	1.10 (0.76, 1.58)	0.60	0.90 (0.54, 1.47)	0.67	0.97 (0.56, 1.66)	0.91

*Note*: Odds ratios (OR) and 95% confidence intervals (CI) were obtained from subtype-specific logistic regression models mutually adjusted for age

*Histologic grade (well differentiated (grade 1), moderately differentiated (grade 2), poorly differentiated (grade 3)), tumor size, lymph nodal involvement, KI67, CK5/6 and EGFR. NE = Not estimated since grade 3 tumors do not qualify as luminal A-like based on the criteria for subtype definition

Within the luminal A-like subtype, the association between p53 expression and parity was stronger when we used >25% staining to define p53 positivity [adjusted OR (95% CI) = 9.07 (1.17, 70.07), *P*-value = 0.03; 9.43 (1.16, 76.51), *P*-value = 0.04; 7.05 (0.77, 64.47), *P*-value = 0.08 for 1, 2, and 3 children, respectively]. However, this analysis was based on a smaller number of cases (*N* = 3746) for whom we had semiquantitative data on p53 IHC. Age did not appear to modify the associations between p53 expression and parity (*P*_interaction_ = 0.59) or breastfeeding (*P*_interaction_ = 0.66) and in analysis stratified by age categories (<50 and ≥50), the findings were similar among younger and older patients (Supplementary Table 2). Also, the results were essentially the same when using cutoff-points of 10 and 20% for ER and PR as compared to those obtained by using a cut-off-point of 1% (Supplementary Table 3). In addition, the mean ER% and PR% expression in the p53+ luminal A-like subtype were 82.7 and 61.7%, respectively, indicating that our findings are unlikely to be due to misclassification of p53+ TNBC as luminal A-like when using a cutoff-point of ≥1% to define ER+ and PR+ tumors.

Since previous studies showed that parity and breastfeeding were differentially associated with luminal A-like and TNBC subtypes; here, we further tested the relationships between parity/breastfeeding and breast cancer subtypes in the context of p53. When using the conventionally used luminal A-like (ER+ and PR+, HER2− and low grade) subtype as the reference group, we found that TNBC patients tended to have increased parity, but the association was not statistically significant. However, when we specifically used the A-like/p53− subtype as the reference group, the parity association became significant for TNBC [adjusted OR (95% CI) vs. nulliparity = 1.82 (1.10, 3.01); *P*-value = 0.02]. In particular, the association was stronger when restricting to TNBC/p53+ patients [adjusted OR (95% CI) vs. nulliparity = 2.39 (1.12, 5.08); *P*-value = 0.02] (Table 5). In contrast, when we used the A-like/p53+ subtype as the reference group, patients with all other subtypes were less likely to be parous or to have breastfed (Table 5), suggesting that the previously observed association between TNBC and parity might be largely driven by p53.

**Table 4:** Adjusted odds ratios and 95% confidence intervals for the associations between epidemiological risk factors and breast cancer subtypes re-classified based on p53 expression

Characteristic	A-like		B-like/HER2−		B-like/HER2+		HER2-enriched		TNBC	
	p53+ vs. p53−		p53+ vs. p53−		p53+ vs. p53−		p53+ vs. p53−		p53+ vs. p53−	
	OR (95% CI)	*P*	OR (95% CI)	*P*	OR (95% CI)	*P*	OR (95% CI)	*P*	OR (95% CI)	*P*
Age at Menarche										
≤12 years	1.00 (reference)		1.00 (reference)		1.00 (reference)		1.00 (reference)		1.00 (reference)	
13 years	1.07 (0.76, 1.50)	0.69	1.36 (0.77, 2.39)	0.29	0.78 (0.40, 1.51)	0.46	1.96 (0.72, 5.30)	0.19	1.12 (0.54, 2.31)	0.76
14 years	0.86 (0.62, 1.19)	0.37	1.44 (0.81, 2.54)	0.21	1.08 (0.56, 2.07)	0.82	1.21 (0.47, 3.15)	0.69	1.25 (0.60, 2.61)	0.54
≥15 years	1.02 (0.76, 1.50)	0.91	1.03 (0.62, 1.70)	0.92	0.74 (0.40, 1.34)	0.32	1.02 (0.42, 2.44)	0.97	0.92 (0.47, 1.78)	0.79
*P trend*		0.99		0.39		0.36		0.33		0.46
Parity										
None	1.00 (reference)		1.00 (reference)		1.00 (reference)		1.00 (reference)		1.00 (reference)	
1	2.67 (1.59, 4.51)	<0.01	0.79 (0.37, 1.67)	0.54	1.06 (0.47, 2.37)	0.89	1.52 (0.49, 4.77)	0.47	1.23 (0.42, 3.58)	0.71
2	2.63 (1.52, 4.55)	<0.01	0.74 (0.34, 1.63)	0.46	0.97 (0.40, 2.33)	0.94	1.43 (0.43, 4.78)	0.56	1.44 (0.47, 4.42)	0.52
≥3	3.67 (2.01, 6.71)	<0.01	1.26 (0.52, 3.04)	0.60	0.82 (0.31, 2.15)	0.68	1.66 (0.38, 7.26)	0.50	0.98 (0.28, 3.45)	0.98
*P trend*		<0.01		0.38		0.42		0.74		0.88
Breastfeed										
Never	1.00 (reference)		1.00 (reference)		1.00 (reference)		1.00 (reference)		1.00 (reference)	
Ever	1.38 (1.03, 1.85)	0.03	0.81 (0.51, 1.29)	0.38	1.18 (0.70, 2.00)	0.54	0.87 (0.37, 2.02)	0.74	0.76 (0.39, 1.49)	0.43
BMI (kg/m^2^)										
≤18.5	0.90 (0.44, 1.85)	0.78	1.29 (0.51, 3.26)	0.59	1.45 (0.48, 4.33)	0.51	1.33 (0.13, 13.4)	0.81	0.51 (0.15, 1.67)	0.27
18.5–24.99	1.00 (reference)		1.00 (reference)		1.00 (reference)		1.00 (reference)		1.00 (reference)	
25–30	1.01 (0.84, 1.23)	0.88	0.79 (0.57, 1.09)	0.16	0.91 (0.63, 1.30)	0.59	0.77 (0.45, 1.32)	0.34	1.01 (0.67, 1.48)	0.96
>30	0.93 (0.67, 1.30)	0.68	0.80 (0.48, 1.35)	0.40	1.01 (0.53, 1.93)	0.98	0.97 (0.36, 2.59)	0.96	0.67 (0.36, 1.22)	0.19
Family history										
Absent	1.00 (reference)		1.00 (reference)		1.00 (reference)		1.00 (reference)		1.00 (reference)	
Present	0.97 (0.69, 1.35)	0.84	0.90 (0.52, 1.56)	0.71	1.72 (0.77, 3.84)	0.19	0.66 (0.26, 1.70)	0.39	1.54 (0.71, 3.34)	0.27

*Note*: Odds ratios (OR) and 95% confidence intervals (CI) were obtained from subtype-specific logistic regression models mutually adjusted for age, age at menarche, parity or breastfeeding, BMI and family history of breast cancer

To test this hypothesis, we modeled parity and breastfeeding as separate outcome variables and p53 expression, breast cancer subtype, CK5/6, EGFR, and other risk factors as explanatory variables. We observed that only p53 positivity [OR (95% CI) = 1.51 (1.10, 2.09); *P*-value = 0.01] was significantly associated with parity. In contrast, the TNBC subtype [OR (95% CI) = 1.52 (0.98, 2.37); *P*-value = 0.06], rather than p53 status, showed the strongest association with breastfeeding (Table 6).

**Table 5:** Adjusted odds ratios and 95% confidence interval for the associations between parity and breastfeeding and breast cancer subtypes overall and following re-classification of the luminal A-like subtype by p53 expression

Subtype	Parous/nulliparous	Parous vs. nulliparous		Ever/never	Ever vs. never breastfed	
		OR (95% CI)	*P*		OR (95% CI)	*P*
A-like (Overall)	2590/114	1.00 (reference)		2049/320	1.00 (reference)	
B-like/HER2−	959/44	0.94 (0.65, 1.35)	0.75	720/127	0.89 (0.71, 1.11)	0.30
B-like/HER2+	775/33	1.17 (0.78, 1.75)	0.45	594/98	1.01 (0.78, 1.29)	0.96
HER2-enriched	386/16	1.15 (0.67, 1.98)	0.61	319/46	1.12 (0.80, 1.57)	0.50
TNBC	681/22	1.36 (0.85, 2.18)	0.20	518/69	1.18 (0.89, 1.57)	0.24
A-like/p53−	1152/63	1.00 (reference)		940/153	1.00 (reference)	
A-like/p53+	858/20	2.67 (1.60, 4.51)	<0.01	637/80	1.38 (1.03, 1.85)	0.03
B-like/HER2− (overall)		1.25 (0.83, 1.87)	0.28		0.96 (0.74, 1.24)	0.74
B-like/HER2−/p53−	295/12	1.35 (0.71, 2.57)	0.35	229/35	1.08 (0.72, 1.61)	0.71
B-like/HER2−/p53+	415/23	1.06 (0.64, 1.75)	0.81	285/58	0.85 (0.61, 1.20)	0.36
B-like/HER2+ (overall)		1.56 (1.01, 2.43)	0.04		1.09 (0.82, 1.44)	0.55
B-like/HER2+/p53−	227/12	1.30 (0.68, 2.48)	0.43	180/32	0.99 (0.65, 1.51)	0.97
B-like/HER2+/p53+	341/16	1.37 (0.77, 2.44)	0.28	249/40	1.14 (0.78, 1.67)	0.50
HER2-enriched (overall)		1.55 (0.88, 2.75)	0.13		1.22 (0.85, 1.75)	0.28
HER2-enriched/p53−	92/6	1.03 (0.42, 2.49)	0.95	83/10	1.45 (0.73, 2.89)	0.29
HER2-enriched/p53+	169/8	1.32 (0.61, 2.84)	0.48	137/18	1.34 (0.79, 2.28)	0.27
TNBC (overall)		1.82 (1.10, 3.01)	0.02		1.28 (0.94, 1.74)	0.12
TNBC/p53−	210/7	1.69 (0.75, 3.79)	0.20	164/15	1.82 (1.03, 3.20)	0.04
TNBC/p53+	317/8	2.39 (1.12, 5.08)	0.02	243/30	1.37 (0.90, 2.09)	0.14
A-like/p53+	858/20	1.00 (reference)		637/80	1.00 (reference)	
B-like/HER2− (overall)		0.47 (0.27, 0.80)	<0.01		0.69 (0.51, 0.94)	0.02
B-like/HER2−/p53−	295/12	0.50 (0.24, 1.05)	0.07	229/35	0.78 (0.51, 1.21)	0.27
B-like/HER2−/p53+	415/23	0.39 (0.21, 0.73)	<0.01	285/58	0.62 (0.43, 0.90)	0.01
B-like/HER2+ (overall)		0.58 (0.33, 1.03)	0.07		0.79 (0.57, 1.09)	0.15
B-like/HER2+/p53−	227/12	0.48 (0.23, 1.02)	0.06	180/32	0.72 (0.46, 1.13)	0.16
B-like/HER2+/p53+	341/16	0.51 (0.26, 1.01)	0.05	249/40	0.83 (0.55, 1.26)	0.38
HER2-enriched (overall)		0.58 (0.29, 1.14)	0.11		0.88 (0.60, 1.31)	0.54
HER2-enriched/p53−	92/6	0.38 (0.15, 0.99)	0.05	83/10	1.06 (0.52, 2.14)	0.88
HER2-enriched/p53+	169/8	0.49 (0.21, 1.15)	0.10	137/18	0.98 (0.56, 1.79)	0.93
TNBC (overall)		0.68 (0.36, 1.26)	0.22		0.93 (0.65, 1.31)	0.67
TNBC/p53−	210/7	0.63 (0.26, 1.53)	0.31	164/15	1.32 (0.74, 2.37)	0.35
TNBC/p53+	317/8	0.89 (0.38, 2.05)	0.78	243/30	0.99 (0.63, 1.57)	0.99

*Note*: Odds ratios (OR) and 95% confidence intervals (CI) were obtained in multivariate polytomous logistic regression models mutually adjusted for parity or breastfeeding, age, age at menarche, BMI, family history of breast cancer

## Discussion

Including over 7000 women with histologically confirmed invasive breast cancer, this large-scale, case-only analysis evaluated the clinical and epidemiological relevance of p53 protein expression in molecular subtypes of breast cancer. Our findings indicate that p53 protein expression can be used to further stratify women with luminal A-like breast cancer into subgroups with clinical and epidemiological relevance.

**Table 6:** Adjusted odds ratios and 95% confidence intervals for the associations between selected tumor clinicopathological characteristics and parity and breastfeeding

Characteristic	Parous/nulliparous	Parous vs. nulliparous		Ever/never	Ever vs. never breastfed	
		OR (95% CI)	*P*		OR (95% CI)	*P*
p53 expression						
p53−	1877/94	1.00 (reference)		1537/234	1.00 (reference)	
p53+	2015/75	1.51 (1.10, 2.09)	0.01	1511/216	1.11 (0.90, 1.36)	0.32
Breast cancer subtype						
Luminal A-like	1937/78	1.00 (reference)		1519/225	1.00 (reference)	
Luminal B-like/HER2−	697/35	0.78 (0.51, 1.22)	0.28	511/92	0.84 (0.63, 1.11)	0.22
Luminal B-like/HER2+	551/28	0.88 (0.55, 1.39)	0.58	421/67	1.02 (0.75, 1.39)	0.88
HER2-enriched	249/14	0.86 (0.45, 1.65)	0.65	211/27	1.31 (0.83, 2.08)	0.24
TNBC	458/14	1.31 (0.65, 2.61)	0.45	386/39	1.52 (0.98, 2.37)	0.06
CK5/6 expression						
CK5/6−	3430/153	1.00 (reference)		2679/406	1.00 (reference)	
CK5/6+	462/16	1.68 (0.90, 3.13)	0.10	369/44	1.36 (0.90, 2.37)	0.14
EGFR expression						
EGFR−	2855/119	1.00 (reference)		2244/333	1.00 (reference)	
EGFR+	1037/50	0.67 (0.43, 1.04)	0.07	804/117	0.77 (0.57, 1.05)	0.10

*Note*: Odds ratios (ORs) and 95% confidence intervals (CIs) were obtained from multivariate logistic regression models separately for parity and breastfeeding that were mutually adjusted for p53, breast cancer subtype, CK5/6 and EGFR, in addition to age, BMI, and age at menarche

The frequency of *TP53* mutation varies according to molecular subtype of breast cancer, with luminal tumors tending to have lower prevalence than basal-like or HER2-enriched tumors.^27–29^ In terms of IHC expression, several cut-points have been proposed to indicate p53+ tumors, including ≥1, 5, 10, or 50%, with reported frequencies ranging from 9 to 54%.^32–38^ In the current study, we used a threshold of ≥1% and observed 53% of the tumors to be p53+, which is similar to the 54% that was reported by van der Kooy and colleagues^33^ when considering any degree of nuclear staining (i.e., ≥1%). At a cut-point of 10%, they observed ~27% of the tumors to be p53+. Due to lack of p53 data on a continuous scale for all patients, we were unable to examine the prevalence of p53+ tumors at the 10% cut-point. However, consistent with other reports,^31^ we observed its frequency to vary by molecular subtype, with the luminal A-like subtype having the lowest frequency and the TN and HER2-enriched having the highest frequencies.

Several reports have documented molecular and clinical heterogeneity within subtypes of breast cancer, which is most notable for the luminal-like subtype.^4,9–11^ Although proliferation markers (KI67, histologic grade) have been used to further stratify luminal-like breast cancer into ‘pure’ A-like, B-like/HER2−, and B-like/HER2+ subtypes, data from recent molecular profiling studies suggest that luminal A tumors were still comprised of prognostically distinct subgroups.^39^ In line with this, a previous study reported that the combination of p53/KI67 provided better prognostic stratification than when KI67 was used alone. In keeping with this notion, we found evidence in support of differences in the clinicopathological features of luminal A-like/p53+ vs. A-like/p53− tumors. We observed that compared with luminal A-like/p53− tumors, A-like/p53+ tumors tended to occur among younger women, to be of higher grade, to have higher levels of proliferation, and to more frequently express basal markers. The precise clinical relevance of this finding remains to be determined, but it is highly suggestive of a role for p53 IHC in further refining luminal A-like breast cancer classification into subgroups with prognostic and, probably, therapeutic implications.

In addition to differences in clinical outcomes, breast cancer subtypes are etiologically heterogeneous.^3,40^ Results from epidemiological studies have shown that reproductive and hormonal risk factors were more consistently associated with the risk of luminal than non-luminal tumors.^3,40^ Interestingly, accumulating evidence is in support of within-subtype heterogeneity according to other clinically relevant tumor characteristics.^41–43^ In this study, we found p53 expression to identify a phenotype of luminal A-like breast cancer that may also be etiologically relevant given its association with parity and breastfeeding. It is unclear why parity/breastfeeding was associated with p53 expression only in luminal A-like tumors. One possible explanation may have to do with differences in the timing of *TP53* mutations in different breast cancer subtypes.^26^ Limited evidence from one study on *BRCA1* mutated breast tumors suggested that in contrast to basal-like tumors in which *TP53* mutations occur late, *TP53* mutations may occur early in the development of luminal A tumors.^26^ Nonetheless, the relatively small number of p53− cases in the other subtypes may have led to reduced power to detect significant differences.

Due to the absence of controls, we were unable to determine the direction of risk for the association between parity/breastfeeding and p53 expression. However, we postulate that the well-established protective effect of parity on luminal breast cancer^40,44^ may be limited to p53− patients, while parity may either not be associated with or may be associated with an increased risk for p53+ tumors in a similar way to TNBC.^5–7^ This hypothesis is consistent with findings from an ovarian cancer study, in which parity was shown to be associated with a decreased risk [OR (95% CI) = 0.31 (0.18, 0.55)] of p53− but not p53+ [OR (95% CI) = 0.92 (0.54, 1.59)] tumors (*p*-value for heterogeneity <0.001).^45^ Indeed, given the high rate of *TP53* mutations in TNBC, our findings raise the prospect that previously observed associations with parity in this subtype may be related to perturbations in the *TP53* pathway. To further buttress this, we observed stronger evidence in support of differences between A-like and TNBC in relation to parity when the A-like/p53− and A-like/p53+ subtypes were modeled separately than when the A-like subtype was treated as a homogeneous entity, as is conventionally done. It has been hypothesized that terminal duct lobular unit (TDLU) involution and the attendant chronic inflammation that accompanies pregnancy may underpin the increased risk of cancer among parous women.^6,46^ Moreover, results from experimental studies have shown a mutual negative regulation of NF-κB, a key regulator of the chronic inflammatory process, and *TP53* function.^47^ It is, therefore, plausible that the association between parity and p53 expression may be due to perturbations in the *TP53* pathway secondary to aberrant post-partum involution and chronic inflammation. In contrast to what has been previously reported for women from Western populations that TNBC patients were less likely to breastfeed than luminal patients,^40,48,49^ our findings are suggestive of a positive relationship between breastfeeding and the luminal A-like/p53+ and TNBC subtypes. Discrepancies may be reflective of population/ethnic differences in the relationship between breastfeeding and breast cancer subtypes. For instance, we^50^ and others^7,51^ have previously shown that the prevalence of breastfeeding was higher among Asian women with the luminal B-like, HER2-enriched, and TN than luminal A-like breast cancer subtype.

To the best of our knowledge, this is the first study to specifically examine the association between breast cancer risk factors and p53 expression in the context of breast cancer subtypes. Based on our findings, we have also suggested a mechanistic framework by which p53 may drive previously observed associations between parity and TNBC. An additional strength of this study was the availability of data on several tumor clinicopathological factors, which enabled us to define subtypes based on current guidelines and to account for correlated tumor characteristics in our analyses. Participants in this study were from a single institution in China with centralized measures on IHC markers thereby limiting the impact of pre-analytical variability on our results.

This study is not without limitations. First, due to lack of information on clinical outcomes, we were unable to directly evaluate the prognostic value of p53 expression in breast cancer subtypes. Owing to the absence of controls, our case-case findings could not be translated into relative risk estimates, which limited the analysis of incorporating p53 expression status in re-defining etiologically relevant subtypes of breast cancer. Second, we used p53 IHC as a surrogate for mutation status. IHC detection of mutant *TP53* is based on its accumulation in the nucleus and its long half-life. Although this method shows reasonable correlation with mutation status in previous studies, it is not a perfect surrogate for complex (deletions or insertions) mutations.^52^ In addition, due to the lack of data on a continuous scale for the majority of our patients, we were unable to evaluate the value of using 10% as the threshold for defining p53+ tumors despite that it is currently the most commonly applied cutoff-point in the literature. Nonetheless, a conservative cut-point of ≥1%, which includes cases with p53 expression of 1–9%, is more likely to lead to under, rather than over, estimation of odds ratios. To buttress this point, we observed the association between parity and p53 expression to be much stronger when we compared women with p53 expression >25% to those with <1% as part of sensitivity analysis. Third, limited power, particularly for minor subtypes, may have hindered our ability to detect clinical and etiological differences by p53 expression within these subtypes. Fourth, we defined subtypes by using IHC markers. Although well-established, dichotomization of the different IHC markers, particularly ER and PR, could lead to subtype misclassifications depending on cutoff-points. To address this issue, we conducted sensitivity analyses using different cutoff-points and obtained similar results, suggesting that our findings are unlikely to be driven by subtype misclassification. Fifth, because this study was based on patients from a Chinese population, the external generalizability of the findings remains unclear. Future studies conducted in different populations with the inclusion of appropriate controls, quantitative measurement of TP53 expression, detailed annotation of clinical outcome and risk factor data are warranted to confirm our findings.

In conclusion, findings from this study are in support of additional clinical and epidemiological heterogeneity within luminal A-like breast cancer that is driven by p53 expression status. Notably, our findings indicate that p53 expression can be used to identify a p53+ luminal A-like breast cancer phenotype that is characterized by younger age at onset, high KI67 proliferation index, high EGFR expression, as well as associations with parity and breastfeeding practices. These clinicopathological and epidemiological attributes are analogous to what have been reported for TNBC, an aggressive subtype of breast cancer. Taken together, these findings suggest that p53 IHC could be used to refine luminal A-like breast cancer definition with clinical and epidemiological implications. Further case-control or prospective cohort studies will be required to confirm the direction of risk for the associations between parity, breastfeeding, and p53 expression in luminal A-like breast cancer.

## Methods

### Study population

This study is a hospital-based case-series comprised of women with histologically confirmed invasive breast cancer who were diagnosed and treated at the Cancer Hospital, Chinese Academy of Medical Sciences (CHCAMS), Beijing, China, between 2009 and 2016. A large sample of 7226 women had p53 IHC information available, as well as relevant tumor clinicopathological characteristics and breast cancer risk factors. Data on histopathological characteristics including histologic grade, lymph nodal involvement, tumor size, ER, PR, HER2, KI67, epidermal growth factor receptor (EGFR), and cytokeratin 5/6 (CK5/6) were obtained from pathology records. This project received ethical approval from the CHCAMS Ethics Committee and was exempted from review by the Office of Human Research Protections at the National Institutes of Health, NIH (exempt number 11751), since it did not involve interaction with human subjects and/or use of individual’s personal identifying information. Informed consent was not required for the use of existing pathological materials with no reveal of identifiable patient information.

### Immunohistochemical staining and scoring

All IHC markers were stained using standard laboratory procedures (supplementary Table 1). Staining was performed using rabbit monoclonal antibodies for ER (SP1 clone, catalog number 790–4325, 1:1000 dilution, Roche), PR (1E2 clone, catalog number 790–4296, 1:1000 dilution, Roche), HER2 (4B5 clone, catalog number 790–4493, 1:166 dilution, Roche), and EGFR (5B7 clone, catalog number 790–4347, 1:500 dilution, Roche). CK5/6, P53, and KI67 were stained using mouse monoclonal antibodies MX040 (catalog number MAB-0276, Maixin), MX008 (catalog number MAB-0142, Maixin), and MIB-1 (catalog number MAB-0129, Miaxin) based on manufacturer optimized concentrations for each antibody. We used the Roche Ventana XT autostainer for all markers, except for CK5/6 and p53 for which we used Dako and Leica autostainers, respectively. All markers were visually assessed by pathologists. For ER and PR, a cut-point of 1% was selected to conform with recommendations by international guidelines.^20^ To test the potential impact of misclassifications, we also redefined subtypes based on ER/PR expression of 10% and 20%. For HER2, a score of 3+ on IHC, or amplification on fluorescence in situ hybridization (FISH), was considered as positive based on the American Society for Clinical Oncology (ASCO)/College of American Pathologists (CAP) guidelines. Cases with HER2 IHC scores 2+ but for whom information on FISH was not available were classified as HER2-negative. Because visual KI67 staining >25% has been shown to provide the best survival discrimination than other cut-points,^53^ cases with KI67 score above this threshold were considered positive. For p53 expression, the majority of the patients had dichotomous categories (0 = negative, 1 = positive). However, a more detailed scoring based on % nuclear staining (<1%; 1–25%; 50–75%; and >75%) was available for a subset of patients. In line with previous studies,^33^ cases with P53 staining ≥1% were considered as positive. EGFR and CK5/6 staining were scored as 0 (negative) and 1 (positive; any staining), similarly as what we used in our previous studies.^54^

### Breast cancer risk factors

Information on breast cancer risk factors were curated from medical records, which included age at diagnosis (*N* = 7226), age at menarche (*N* = 4697), parity (*N* = 4670), breastfeeding practices (*N* = 3997), body mass index (BMI, *N* = 4719), and family history of breast cancer in a first degree relative (*N* = 4776).

### Breast cancer subtype definition

Molecular subtypes were defined based on the St Gallen’s criteria by using IHC measures on ER, PR, and HER2 in conjunction with histologic grade.^16,18^ Luminal A-like: ER+ and PR+, HER2− and low grade (grades 1 or 2); Luminal B-like/HER2−: ER+ and/or PR+, HER2− and high grade (grade 3); Luminal B-like/HER2+: ER+ and/or PR+, HER2+ (regardless of grade); HER2-enriched: ER−, PR−, and HER2+; Basal-like/TN: ER−, PR−, HER2−. All the breast cancer subtypes were further re-classified using p53 expression.

### Statistical analysis

Frequency tables were used to assess the distribution of tumor clinicopathological features (tumor size [<2 cm (reference), 2–5 cm, and >5 cm], lymph node involvement [0 (reference), 1–3, and >3], and histologic grade [well-differentiated (reference), moderately differentiated, and poorly differentiated]) and epidemiological risk factors (age [<40 (reference), 40–50, 50–60, 60–70, and ≥70], age at menarche [in years: ≤12 (reference), 13, 14, and ≥15], parity [nulliparous (reference), 1, 2, and ≥3 children], breastfeeding [never (reference), ever], BMI [in kg/m^2^, based on World Health Organization (WHO) categories^55^: <18.5 (underweight); 18.5–24.99 (normal, reference); 25–30 (overweight); and >30 (obese)], and family history of breast cancer in first-degree relatives [absent (reference), present].

We used the chi-square test to assess differences in the distribution of categorical variables by p53 expression status and the nonparametric Kruskal-Wallis test for continuous variables. Odds ratios (ORs) and 95% confidence intervals (CIs) for the associations of p53 expression with breast cancer risk factors and tumor clinicopathological features were estimated in age-adjusted unconditional logistic regression models. To assess these associations within each breast cancer subtype, we constructed logistic regression models with subtype-p53 status [for example, luminal A-like/p53-positive (A-like/p53+) vs. luminal A-like/p53-negative (A-like/p53−)] as the outcome and each risk factor or clinicopathological factor (adjusted for age) as the explanatory variable. We also conducted multivariate regression analyses by including all examined risk factors in the model. To determine which of the variables among p53 expression, TNBC subtype, or basal marker expression i.e., CK5/6 and EGFR, was predictive of parity, we modeled parity as the outcome (parous vs nulliparous) and included p53 (p53+ vs. p53−), breast cancer subtypes, CK5/6 (CK5/6+ vs. CK5/6−), EGFR (EGFR+ vs. EGFR−), in addition to age, age at menarche, and BMI, as explanatory variables.

We adopted several strategies for sensitivity analyses. First, we performed the risk factor analysis for women stratified into two age groups (<50 years and ≥50 years). Second, we substituted p53 with basal markers CK5/6 and/or EGFR in molecular subtypes and repeated the risk factor analysis. Third, we substituted grade with KI67 to define the luminal A-like and luminal B-like/HER2− subtypes and repeated all analyses using these subtypes. Fourth, we defined p53+ tumors using a threshold of >25% and examined associations with risk factors. Fifth, we examined the associations between clinical and epidemiological factors with p53 expression in luminal A-like subtype defined based on 10 and 20% cut-points for ER/PR. Since we found no evidence of modification of our results by age or by basal markers, and because the results were essentially the same regardless of whether subtypes were defined by grade or KI67, results presented in tables were for all age groups and for subtypes defined by grade. Missing values on risk factor covariates were addressed using the listwise deletion approach. In additional sensitivity analyses, we created indicators for missing values on each risk factor in the multivariate regression model and our results remained essentially the same. All analyses were two-sided and conducted using Stata statistical software version 14.1.

### Reporting summary

Further information on research design is available in the Nature Research Reporting Summary linked to this article.

## Supplementary information

Supplementary Information

Reporting Summary

## Data Availability

The data that support the findings of this study belong to the Cancer Hospital, Chinese Academy of Medical Sciences (CHCAMS) which granted the approval for use in this analysis. Due to other ongoing clinical and epidemiological research within the same data, restrictions apply to the use of these data and so are not made publicly available, but these data are available from the corresponding author upon reasonable request without undue qualification. The data generated and analyzed during this study are described in detail in the following data record^56^: 10.6084/m9.figshare.8300135.

## Supplementary information

**Supplementary Information** accompanies the paper on the *npj Breast Cancer* website (10.1038/s41523-019-0117-7).

## Code availability

The codes that support the findings of this study are available from the corresponding author upon reasonable request without undue qualification.
