# Supplementary material for: Clinicopathological and epidemiological significance of breast cancer subtype reclassification based on p53 immunohistochemical expression
Source: NPJ Breast Cancer. 2019 Jul 25;5:20. doi: 10.1038/s41523-019-0117-7 (PMC6658470; doi:10.1038/s41523-019-0117-7)
Supplement: Supplementary file 1 — Supplementary Information [file 41523_2019_117_MOESM1_ESM.pdf]

Supplementary Table 1. Details of antibodies and staining equipment for immunohistochemical stains

Antibody	Clone	Manufacturer	Catalogue number	Stainer
ER	SP1 （rabbit monoclonal）	Roche	790-4325	Roche Ventana XT
PR	1E2 （rabbit monoclonal）	Roche	790-4296	Roche Ventana XT
HER2	4B5 （rabbit monoclonal）	Roche	790-4493	Roche Ventana XT
EGFR	5B7 （rabbit monoclonal）	Roche	790-4347	Roche Ventana XT
Ki67	MIB-1 (mouse monoclonal)	Maixin	MAB-0129	Roche Ventana XT
CK5/6	MX040 （mouse monoclonal）	Maixin	MAB-0276	Dako Autostainer Link 48
TP53	MX008 （mouse monoclonal）	Maixin	MAB-0142	Leica RM2235

Supplementary Table 2. Odds ratios and 95% confidence intervals for the associations between breast cancer risk factors and phenotypes (p53+ vs p53-) of luminal A-like breast cancer among younger (≤50 years) and older women (>50 years)

	≤50 years		>50 years	
	p53+ vs p53-		p53+ vs p53-	
**Characteristic**	**OR (95% CI)**	**p-value**	**OR (95% CI)**	**p-value**
Age at Menarche				
≤12 yrs.	1.00 (reference)		1.00 (reference)	
13 yrs.	0.82 (0.52, 1.27)	0.37	1.60 (0.94, 2.75)	0.08
14 yrs.	0.69 (0.45, 1.05)	0.09	1.23 (0.73, 2.09)	0.43
≥15 yrs.	0.95 (0.64, 1.40)	0.79	1.29 (0.80, 2.08)	0.29
p_trend		0.89		0.97
Parity				
None	1.00 (reference)		1.00 (reference)	
1	2.07 (1.16, 3.70)	0.01	15.23 (2.03, 114.42)	0.01
2	2.36 (1.25, 4.47)	0.01	13.22 (1.75, 99.91)	0.01
≥3	2.22 (0.95, 5.12)	0.06	20.21 (2.63, 155.31)	<0.01
p_trend		0.06		0.06
Breastfeed				
Never	1.00 (reference)		1.00 (reference)	
Ever	1.34 (0.91, 1.98)	0.14	1.47 (0.92, 2.33)	0.10
BMI, kg/m2				
≤18.5	1.07 (0.43, 2.68)	0.88	0.67 (0.20, 2.22)	0.51
18.5-24.99	1.00 (reference)		1.00 (reference)	
25-30	1.04 (0.79, 1.38)	0.77	1.01 (0.77, 1.32)	0.93
>30	1.07 (0.64, 1.79)	0.78	0.87 (0.56, 1.34)	0.52
Family History				
Absent	1.00 (reference)		1.00 (reference)	
Present	1.08 (0.67, 1.75)	0.73	0.82 (0.51, 1.32)	0.42

Odds ratios and 95% confidence interval were obtained from subtype-specific logistic regression models mutually adjusted for age, age at menarche, parity, breastfeeding, BMI and family history of breast cancer.

Supplementary Table 3. Adjusted odds ratios and 95% confidence intervals for the associations between breast cancer clinicopathological and epidemiological factors and p53 expression in luminal A-like breast cancer defined using different cutoff-points for ER and PR expression

	Luminal A-like (1% ER and PR)		Luminal A-like (10% ER and PR)		Luminal A-like (20% ER and PR)	
	p53+ vs p53-		p53+ vs p53-		p53+ vs p53-	
**Characteristic**	**OR (95% CI)**	**p-value**	**OR (95% CI)**	**p-value**	**OR (95% CI)**	**p-value**
Age, yrs.						
<40	1.00 (reference)		1.00 (reference)		1.00 (reference)	
40-50	1.04 (0.81, 1.32)	0.76	1.12 (0.87, 1.44)	0.38	1.07 (0.80, 1.44)	0.62
50-60	0.83 (0.65, 1.06)	0.14	0.84 (0.65, 1.09)	0.19	0.78 (0.58, 1.06)	0.11
60-70	0.78 (0.59, 1.03)	0.08	0.83 (0.62, 1.11)	0.21	0.76 (0.55, 1.06)	0.11
70+	0.62 (0.44, 0.88)	<0.01	0.72 (0.50, 1.04)	0.08	0.78 (0.51, 1.19)	0.24
p_*trend*		<0.01		0.03		0.04
Histologic grade						
Well diff.	1.00 (reference)		1.00 (reference)		1.00 (reference)	
Mod. diff.	1.69 (1.35, 2.12)	<0.01	1.41 (1.11, 1.80)	<0.01	1.32 (1.00, 1.73)	0.05
Tumor size						
<2cm	1.00 (reference)		1.00 (reference)		1.00 (reference)	
2-5cm	1.18 (0.95, 1.47)	0.13	1.01 (0.80, 1.27)	0.91	1.20 (0.90, 1.59)	0.21
>5cm	1.14 (0.57, 2.27)	0.71	0.90 (0.44, 1.84)	0.77	0.87 (0.37, 2.01)	0.75
p_*trend*		0.15		0.95		0.26
Nodal Involvement						
0	1.00 (reference)		1.00 (reference)		1.00 (reference)	
1-3	0.99 (0.84, 1.17)	0.92	0.94 (0.79, 1.12)	0.49	0.94 (0.76, 1.15)	0.53
>3	1.16 (0.95, 1.40)	0.14	1.10 (0.89, 1.35)	0.37	1.09 (0.87, 1.39)	0.45
KI67						
Q1 (<10)	1.00 (reference)		1.00 (reference)		1.00 (reference)	
Q2 (10-20)	1.28 (1.05, 1.55)	0.02	1.32 (1.07, 1.62)	<0.01	1.38 (1.07, 1.79)	0.01
Q3 (20-35)	1.77 (1.45, 2.14)	<0.01	1.78 (1.44, 2.19)	<0.01	1.97 (1.53, 2.55)	<0.01
Q4 (>35)	2.47 (1.88, 3.24)	<0.01	2.43 (1.82, 3.24)	<0.01	2.49 (1.77, 3.51)	<0.01
p_*trend*		<0.01		<0.01		<0.01
CK5/6						
Negative	1.00 (reference)		1.00 (reference)		1.00 (reference)	
Positive	1.55 (0.85, 2.80)	0.15	0.91 (0.45, 1.85)	0.80	1.38 (0.58, 3.26)	0.46
EGFR						
Negative	1.00 (reference)		1.00 (reference)		1.00 (reference)	
Positive	1.95 (1.41, 2.69)	<0.01	1.79 (1.24, 2.57)	<0.01	1.77 (1.18, 2.67)	<0.01
Age at Menarche						
≤12 yrs.	1.00 (reference)		1.00 (reference)		1.00 (reference)	
13 yrs.	1.07 (0.76, 1.50)	0.69	1.08 (0.76, 1.53)	0.65	1.05 (0.73, 1.52)	0.78
14 yrs.	0.86 (0.62, 1.19)	0.37	0.85 (0.60, 1.18)	0.33	0.83 (0.58, 1.18)	0.30
≥15 yrs.	1.02 (0.76, 1.50)	0.91	1.01 (0.75, 1.37)	0.92	1.06 (0.77, 1.45)	0.72
p_*trend*		0.99		0.98		0.68
Parity						
None	1.00 (reference)		1.00 (reference)		1.00 (reference)	
1	2.67 (1.59, 4.51)	<0.01	2.88 (1.67, 4.96)	<0.01	3.23 (1.80, 5.81)	<0.01
2	2.63 (1.52, 4.55)	<0.01	2.93 (1.66, 5.17)	<0.01	3.40 (1.85, 6.27)	<0.01
>3	3.67 (2.01, 6.71)	<0.01	3.94 (2.10, 7.40)	<0.01	4.58 (2.34, 8.99)	<0.01
p_*trend*		<0.01		<0.01		<0.01
Breastfeed						
Never	1.00 (reference)		1.00 (reference)		1.00 (reference)	
Ever	1.38 (1.03, 1.85)	0.03	1.46 (1.08, 1.97)	0.01	1.66 (1.20, 2.30)	<0.01
BMI						
≤18.5	0.90 (0.44, 1.85)	0.78	1.02 (0.47, 2.22)	0.95	1.00 (0.43, 2.33)	0.99
18.5-24.99	1.00 (reference)		1.00 (reference)		1.00 (reference)	
25-30	1.01 (0.84, 1.23)	0.88	1.00 (0.82, 1.22)	0.96	1.05 (0.85, 1.30)	0.62
>30	0.93 (0.67, 1.30)	0.68	0.88 (0.63, 1.24)	0.47	1.00 (0.70, 1.42)	0.99
Family History						
Absent	1.00 (reference)		1.00 (reference)		1.00 (reference)	
Present	0.97 (0.69, 1.35)	0.84	0.95 (0.67, 1.34)	0.75	0.91 (0.63, 1.32)	0.62

Odds ratios and 95% confidence intervals were from mutually adjusted multivariate polytomous logistic models separately for clinicopathological features and epidemiological factors.
